# How does national development zone policy affect carbon emissions in China? New evidence from a quasi-natural experiment

**DOI:** 10.3389/fpubh.2023.1122139

**Published:** 2023-04-06

**Authors:** Yanchao Feng, Yue Gao, Yuehua Zhu, Shilei Hu

**Affiliations:** ^1^Business School, Zhengzhou University, Zhengzhou, China; ^2^School of Economics and Management, Harbin Institute of Technology, Weihai, China

**Keywords:** national development zone policy, carbon emissions, spatial difference-in-differences model, quasi-natural experiment, spatial heterogeneity

## Abstract

The expansion of China's development zones has made great contributions to economic development, as well as provided practical guidance for other developing countries to implement development zone policies. However, in the context of global advocacy of low carbon, literature about how the development zone policy affect carbon emissions is poor, especially in China at the urban level. Therefore, this study takes China's development zone policy as a quasi-natural experiment, using the panel data of 285 cities in China from 2003 to 2020, and adopting the DID model to analyze its impact on carbon emissions. After a series of robustness tests including placebo test, dynamic test (all independent variables are lagged by one period), endogeneity test, and parallel trend test, the results are basically robust. The findings show that the development zone policy indeed significantly reduces carbon emissions. In addition, we find that cities with higher resource endowments, cities in the eastern and central regions, and other larger cities across the country have better carbon emissions reduction effects. To a certain extent, the research in this paper fills the gap of theoretical research on carbon emissions in terms of the development zone policy, and provides some practical basis for future research in the field of carbon emissions.

## 1. Introduction

The world's first special economic zone can be traced back to 1959, when the development zone in Shannon, Ireland, was established. Establishing the development zone has brought prosperity to the Irish economy (1). Since then, many countries worldwide have established various special economic zones similar to the development zone [(2–5); Frick et al., 2022]. In particular, China's first batch of national-level development zones was launched in 1984, which has rapidly promoted economic growth due to its unique advantages in management mechanisms, innovative elements, and preferential policies (6). It is widely known that China has the second largest economic system in the world (7), while there is no consensus on the factors driving sustainable economic development, especially in the field of policy evaluation (8). In fact, it is commonly known that national development zone strategy promotes economic development (9). The global economy has slumped in recent years due to the COVID-19 pandemic, and China's economy has also been significantly impacted at the same time, thus the outlook for export commerce situation is not promising. Fortunately, since China's population base is large and is located in a development zone, the Chinese people can alleviate the effects of the pandemic there, allowing China's economy to continue to grow steadily despite the ongoing decline of the world economy (10–12).

Despite the fact that China has experienced significant economic growth in the recent decades (13), the rise in carbon emissions year after year casts doubt on this broad progress (14). China now ranks among the countries with the highest global carbon emissions (15). In contrast, China's carbon emissions in 2017 made up 28% of all carbon emissions worldwide (16). In addition, global carbon dioxide emissions increased in 2021 compared to 2020 by 4.8% (17). Global warming, caused by the recent rapid surge in carbon emissions, poses a serious threat to human sustainability (18). Therefore, the main challenge facing the Chinese government is to find solutions to balance the issues of economic development and environmental conservation (19). Against this background, in order to accept the international duty and foster the development of “a community with a shared destiny for mankind”, Chinese President Xi proposes the program of “strive to peak carbon dioxide emissions by 2030 and strive to achieve carbon neutrality by 2060” in September 2020 (20).

The available literature on the effects of national development zone policy may generally be split into three categories including economy, society, and environment. As for the economy, the development zone has a significant economic impact on the city's technological innovation (21), and it may foster the growth of high-tech enterprises, encourage investment benefits, and broaden the region's economic base (22). In terms of social welfare, the development zone's economic expansion will unavoidably encourage regional growth, raise residents' quality of life, and further the completion of sanitary infrastructure (23). In addition, the level of employment, wealth, and happiness of the populace will rise locally (24). However, the development zone resembles an industrial cluster in terms of ecology. Sulfur dioxide, nitrogen dioxide, and industrial waste water, will be released as a result of mass production, which will take a lot of energy and fuel and which will cause serious damage to the ecological environment (25). At this time, the research how development zone policies affect carbon emissions is limited, which forms the initial incentive of this study, that is, to fill the research gap.

Has the implementation of the development zone policy reduced total carbon emissions? Evidence from a quasi-natural experiment in China. Research on the impact of development zone policies on carbon emissions based on DID model; Study the lagging effect of development zone policies; Analyze whether the effect of implementing development zone policies in different regions, cities of different sizes and different resource types in China is consistent; Study the spatial effects of development zone policies between pilot and non-pilot cities. Through the above correlation analysis, the mechanism of how the implementation of development zone policies and similar policies affects the total carbon emissions is systematically elaborated. It aims to provide guiding advice to developing countries like China. According to existing research findings, the DID model and its derivatives are frequently employed in the field of policy assessment due to their benefits in avoiding issues with endogeneity and omitted variable bias (26, 27). Due to China's unique characteristics, including its huge landmass and wealth of natural resources (28), there may be disparities in the extent of the effects of the development zone policy's implementation in various areas and cities. Additionally, the city's resource endowment will influence how the development zone policy performs (29). Moreover, this work takes into account the effect of policy upgrading or superposition on carbon emissions in order to further illustrate the research scenario (30). In order to examine the effects of development zone policies on urban carbon emissions, this study seeks to arrange development zone policies as a collection of quasi-natural experiments, and employs the DID model as a benchmark regression approach (31). The propensity score matching DID model (PSM-DID model) (32) is used to around the DID model's limitations. Furthermore, the spatial difference model (SDID) is also employed to detect the probable existence of spatial spillover effects (33).

Two characteristics of this study can be used to infer its key contributions (33). Theoretically, research on the effects of development zone rules now focuses majorly on the effects on the economic scale and economic development of the city, while the research on the impacts of the policies on the urban environment is uncommon. In order to conceptually fill the gap left by the absence of this module. We put the development zone policy and carbon emissions into a research framework, and comprehensively analyzed the relationship between the development zone policy and carbon emissions from both static and dynamic perspectives by using various methods. This research has significant worldwide guiding relevance for other emerging nations as well as significant practical meanings for the creation and optimization of development zones including high-tech zones and economic development zones, etc. Practically, this study divided the entire sample into subgroups according to geographic location, urban scale, and resource endowment of the city, which enriches the application value of policy recommendations.

The remainder of the study is organized as follows to maintain its integrity. Section 2 presents and summarizes earlier related studies in a concise manner, and constructs the theoretical mechanism. Section 3 describes the economic model and related variables. Section 4 examines the empirical results and some robustness tests. Section 5 covers the diversity of city location, scale and resource donation, and tests the SDID model and policy upgrading and superposition effects. Section 6 summarizes the research results, offers guidance, and identifies future directions for further study.

## 2. Literature review and theoretical mechanism

The literature review is divided into four sections: The first part provides an overview of the current literature on carbon emissions; The second part analyzes the literature on the economic effects of development zone policies; The third part provides a brief summary of the research literature on the environmental effects of development zone policies; The forth part, through the research of relevant literature, the theoretical mechanisms of the development zone policy's effect on carbon emission are sorted out.

### 2.1. Literature on carbon emissions

As is common knowledge, energy use and carbon emissions are intimately correlated with human activity. Especially, population mobility has a major negative impact on carbon emissions in regions where people live (34, 35). Mobility of the population will unavoidably result in frequent use of transportation, which uses a lot of fossil fuels and raises carbon emissions. In the transportation sector, intelligent mobility can significantly reduce carbon emissions (36). Economic manufacturing will raise carbon emissions in cities, which is another significant element affecting China's industrial development. Some academics contend that while economic progress aids in the rationalization of industrial structure and carbon emissions, the upgrading and optimization of industrial structure has a detrimental effect on carbon emissions (19, 37).

China has a significant agricultural sector, thus its influence on carbon emissions cannot be understated. Research on the steadily rising rural carbon emissions in China's provinces discovered that the provinces closest to the ministry created the most emissions (38). Numerous academics have studied how the use of science and technology in diverse areas influences carbon emissions in light of China's tremendous advancement. Some academics state that China's total carbon emissions are somewhat increasing due to low innovation efficacy of green technology (39, 40). Also, some scholars think that the advancement of green technology benefits the research and creation of renewable resources. Eventually, it has a major detrimental influence on carbon emissions, but has less of an effect in the short term (41). Science and technology advancements encourage the development of digital technology, and these advancements can decrease carbon emissions as a result of their positive knock-on effects (42). According to the study, China's embedded carbon emissions from 2002 to 2017 were significantly impacted negatively by factors relating to the production structure of the digital economy (43).

### 2.2. Literature on the influence of development zone policy on the economy

We can assume that when we talk about the development zone, we are talking about development-related information. Therefore, the focus of this research material is on the development and economic development zones, as well as on how these development zones impact affect the local economy. According to some academics, the creation of development zones can, in some cases, encourage industrial agglomeration, boost industrial productivity, and boost exports. Also, the upgrading strategy policy of development zones influences both imports and exports (44). Moreover, China's development zones have a variety of repercussions, the most notable of which is a considerable impact on nearby manufacturing businesses (45). The creation of the economic development zone serves as a foundation for improving the organization of the industrial land, maximizing its use, and providing a solid land guarantee for the industrial transfer of the manufacturing industry (46).

Development zones can boost investment effectiveness, increase financial openness, and enhance the region's overall economic growth in terms of fostering regional economy (47). Development initiatives strategies has considerably significant spillover and driving impacts and serves as a launchpad for communities to pursue innovation-driven development initiatives (46). Over time, the spillover impacts will differ in numerous ways, largely depending on the special zone's strategic development and policy objectives (48). By distributing resources fairly, the spillover effect can effectively encourage regional economic growth, and help close the economic development gap between regions (22). By maximizing the arrangement of public service facilities, the high-tech zone diversifies urban public service goals in order to better the city as a whole (49). At the macro level, it encourages regional economic growth, and at the micro level, it benefits business performance and the improvement of individual performance (50, 51).

### 2.3. Literature on the impact of development zone policies on the environment

Development zones influence our ecological environment to varied degrees in addition to having an effect on our economic development. Theoretically, the establishment of the development zone will attract a large number of industries to settle in these cities, since industrial manufacturing is an indispensable real economy in a city. Unfortunately, it contaminated the environment of the city in the process of creating the economy. According to Guo et al. (52), the industrial pollution index records an initial decline and a gradual rise with time. Furthermore, the research shows that industrial production will produce a large amount of polluting gases, the most important of which are sulfur dioxide and nitrogen dioxide (52). Simultaneously, the production of heavy industry produces a large amount of industrial wastewater. Palani et al. (53) explain that improper treatment of wastewater will cause pollution of water resources, and the long-term consequence is the decline in the quality of the ecological environment.

Arguably, developing digital industries and sustainable industries are critical skills that can be used to solve this problem. Some researchers hold that developing the digital economy will significantly reduce the industrial pollution (35). The argument is that the high-tech sector puts emphasis on progressing science and technology (54). Consequently, the upgrading of science and technology will improve the efficiency and quality of environmental pollution treatment (55). Because of the varying stages of development zones, the pollution situation after implementation is also very different. The formation of provincial-level development zones further aggravated China's contamination intensity, but rising to the national level, they no longer play the same role (56). Likewise, concerning the smog pollution in the nation, the provincial development zone policies significantly increased smog pollution. On the other hand, the national development zone policies have no significant effect (57). Contrasting the development zones, the establishment of high-tech zones can improve the environment, and the increase of green patents and economic agglomeration are the mechanisms by which high-tech zones can improve the environment (47).

### 2.4. Theoretical mechanism

From the perspective of technological innovation, on the one hand, the establishment and construction of the development zone will attract a large number of high-tech enterprises (22), and at the same time, it will encourage local enterprises to carry out technological innovation activities and increase investment in innovative scientific research (26). High and new technology will improve the efficiency of production and manufacturing, meanwhile, it will increase the utilization rate of raw materials, promoting production efficiency and reducing the consumption of production resources and power resources (58), to achieve the effect of reducing carbon dioxide emissions. On the other hand, the establishment of the development zone will promote the development of emerging green industries such as high-end equipment manufacturing, new energy industries, and lead the direction of urban industrial transformation (59). With the acceleration of the industrial structure upgrading process led by the establishment of the development zone, the living space of the traditional “three high” industries in the city has been further reduced, and the vacated development space will be more occupied by strategic emerging green industries, which makes pollution-free, Clean and green production factors have been widely gathered and applied (60), it can also reduce carbon dioxide emission intensity and improve urban air quality.

From the perspective of resource allocation, on the one hand, the lower land price inside the development zone and related policy grants enable enterprises in the park to enjoy low supply of factors and ensure the continuity of normal production activities of enterprises (13, 61), to promote the efficiency of enterprise factor utilization. In addition, the complete infrastructure of the national development zone has successfully attracted foreign high-quality innovation capital, creating conditions for the emergence and development of high-end productive services (61). On the other hand, the high-end production factors attracted by the development zone can fully replace traditional production factors, promote the development of resource-intensive industries, and gradually reduce environmental pollution (62). To sum up, the optimization of resource allocation caused by the establishment of national development zones will help reduce undesired outputs in the industrial production process (63), and accordingly reduce carbon dioxide emissions.

From the perspective of the city's own conditions, under the background of the establishment of national development zones, there are large differences between different cities in terms of economic scale, innovation factor agglomeration capacity, resource allocation efficiency, and industrial policy formulation and implementation capacity (46). Generally speaking, regional economic development lags behind that of central cities (64). Under the guidance of the construction of ecological civilization, compared with other cities, the central city with stronger agglomeration ability of innovation elements can give full play to its advantages in terms of policy pilot, economic development scale and innovation element agglomeration, so as to achieve the transformation of economic development model (65). And the first-mover advantage of industrial structure adjustment, promote the full release of the potential of urban environmental improvement (66). Due to the relatively poor elements to promote the upgrading of the industrial structure and the poor external environment for industrial development, other cities have great potential and latecomer advantages in terms of environmental improvement capabilities (67). Through the construction of national-level development zone, such cities can obtain the technological spillover effect and high-end production factor agglomeration associated with the establishment of development zones, and fully release the vitality of urban industrial transformation (68), thereby enhancing the city's ability to improve environmental quality by adjusting its industrial structure.

**Figure 1:**
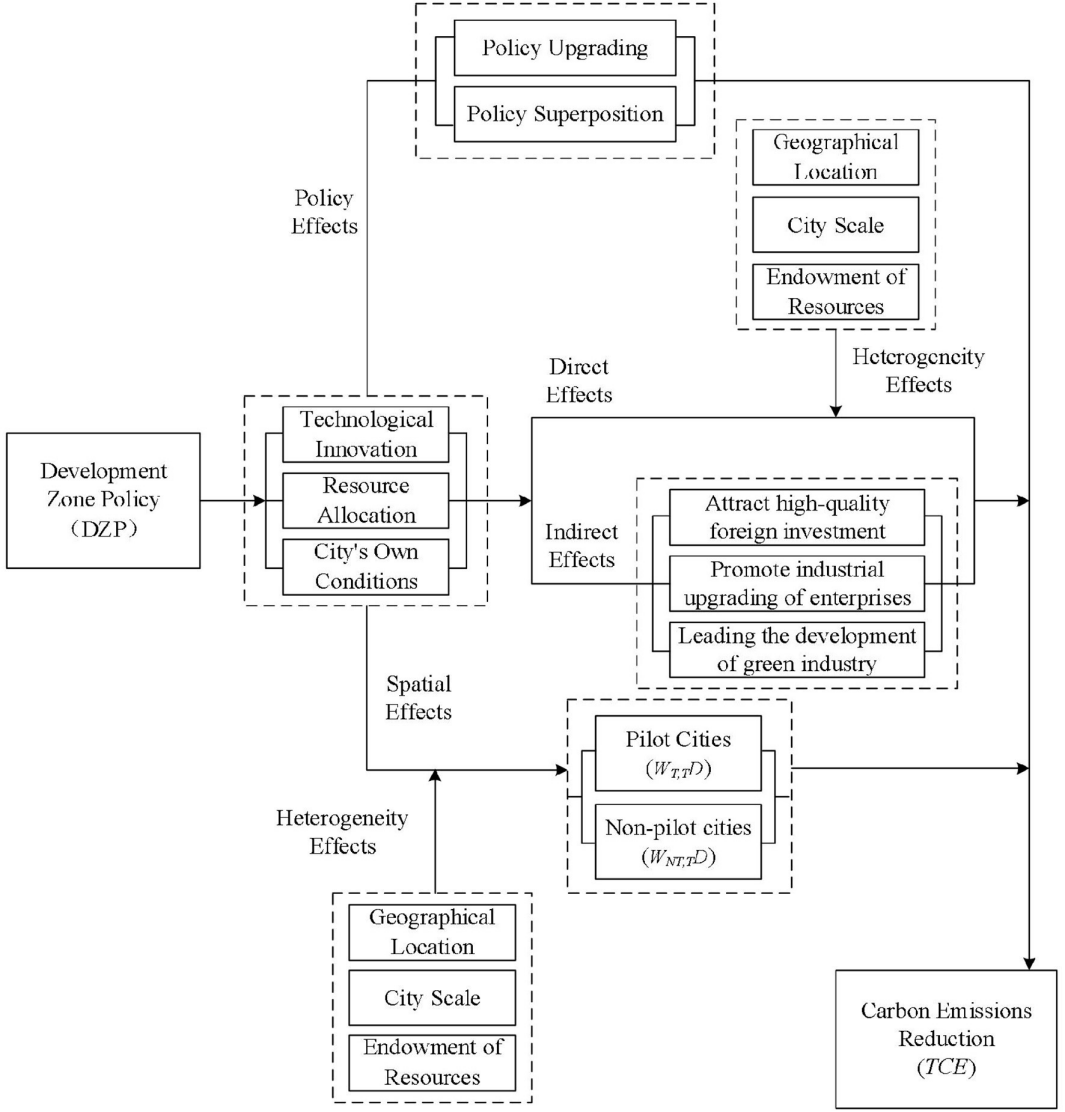
Flowchart of theoretical mechanism.

From the perspective of spatial effects, in view of the spatial agglomeration and spatial differences in my country's environmental policies, institutional environment and energy structure, when examining the impact of the establishment of national development zones on local carbon dioxide emissions, the carbon emissions of neighboring cities should also be taken into account (69). In other words, we should fully consider the spatial spillover effect of policy. With the rapid development of country's digital information technology, cloud computing platform, and big data applications, industries in different cities are more closely connected (70). The development zone policy can not only rely on the transformation of the local energy structure to enhance the green total factor productivity and improve the quality of the urban environment (63), but also have a significant impact on the carbon dioxide emissions of adjacent cities (71). Namely, although the local carbon dioxide emissions have been reduced, their cost may be higher energy consumption in neighboring cities and lower urban environmental quality. In particular, to reveal the theoretical mechanism vividly, we have draw the framework of empirical steps and reported in Figure 1.

## 3. Empirical model

### 3.1. Empirical framework

After the creation of the initial group of development zones, the nation's policies have always maintained development as an essential objective. Pilot areas of development zones across China were the large-scale cities. It is clear that the development brought to the cities caused economic growth and betterment of science and technology. Consumption of resources is another causal factor for economic growth, the consumption of resources is at the expense of the environment, which will bring large emissions of greenhouse gases, such as the total carbon emissions of cities. Research holds that the development of science and technology can ease this occurrence. Theoretically, the use of advanced technology will lower the total carbon emissions of the city. In this regard, this paper considers development zone policies as a set of quasi-natural experiments. The paper also relies on the DID model to assess how development zone policies impact carbon emissions. For this research, pilot cities were listed as the experimental group while the non-pilot cities were the control group. Since the development zone pilots are conducted in different years, a multi-phase DID model will be used. The following is the formula for the multi-period DID model:


(1) $$\begin{array}{l} TCE_{it}=\alpha_{0}+\alpha_{1}DZP_{it}+\beta X_{it}+\lambda_{i}+\lambda_{t}+\mu_{it} \end{array}$$

Where the subscripts *i* and *t* represent city and year, *TCE* is the total carbon emissions of the city, *DZP* represents the dummy variable about the status of the policy implementing, *X* is a series of control variables, λ_*i*_ denotes the individual effect, λ_*t*_ denotes the time fixed effect, α_1_ is the DID estimator, indicating the net effect of policy on total carbon emissions, β is the coefficient of control variables, μ is the random disturbance term, α_0_ is the constant term. *DZP*_*it*_ is the dummy variable, representing a city's development zone policy implementing status. More specifically, *DZP*_*it*_= 1 if city *i* implemented the development zone policy during the sample period, and 0 otherwise.

In addition, we constructed a PSM-DID model for correlation robustness checks because the DID model is not ideal in addressing the problem of sample selection bias. In addition, the model can make the more actual test in line with the theory. Using the model, we can more accurately measure if development zone policies can effectively affect the city's total carbon emissions. The specific theoretical model is as follows:


(2) $$\begin{array}{l} TCE_{it}^{PSM}=\alpha_{0}+\alpha_{1}DZP_{it}+\beta X_{it}+\lambda_{i}+\lambda_{t}+\mu_{it} \end{array}$$

Furthermore, a spatially extended form of the DID model to SDID model based on Equation (1) was used to analyze the spatial spillover effects of policy further. The following is the formulated form of this extended model:


(3) $$\begin{array}{l} \begin{array}{c} TCE_{it}=\alpha_{0}+\alpha_{1}DZP_{it}+\beta X_{it}+\beta_{1}\times W_{T,T}D_{it}\\ +\beta_{2}\times W_{NT,T}D_{it}\\ +\beta_{3}\times W\times X_{it}+\lambda_{i}+\lambda_{t}+\mu_{it} \end{array} \end{array}$$

Where *W* is the spatial weight matrix, *W*_*T, T*_*D*_*it*_represents the spatial spillover effects among pilot cities, *W*_*NT, T*_*D*_*it*_designates the spatial spillover result of pilot cities on non-pilot cities which neighbor pilot cities, *W*×*X*_*it*_ is the spillover effects of control variables. β_1_ is the spatial coefficient of *W*_*T, T*_*D*_*it*_, β_2_ is the spatial coefficient of *W*_*NT, T*_*D*_*it*_, and β_3_ is the spatial coefficient of *W*×*X*_*it*_.

Last but not least, it should be pointed out that the DID model satisfies the parallel trend test. In other words, levels of carbon emission from pilot and non-pilot cities must not differ systematically over time. According to the approach of Jacobson and Sullivan (72), by constructing a series of temporal dummy variables, a temporal analysis research framework is used to analyze the dynamic influence of the application of development zone policies on urban carbon emissions. Consequently, we create a dynamic analysis model as formulated below:


(4) $$\begin{array}{l} TCE_{it}=\alpha_{0}+\overset{t\text{+5}}{\underset{t\text{-}5}{\sum }}\alpha_{t}DZP_{it}+\beta X_{it}+\lambda_{i}+\lambda_{t}+\mu_{it} \end{array}$$

Among them, *DZP* represents the dummy variable of the years before and after the realization of the development zone policy. *DZP*_0_ is the dummy variable of the year when the city enforces the development zone policy; *DZP*_*t*−*n*_ is the dummy variable of *n* years before the implementation of the development zone policy; *DZP*_*t*+*n*_ is the dummy variable of *n* years after the enforcement of the development zone policy; other variables are constant as in the case of Equation (1).

### 3.2. Data and variables

Panel datasets and IPE research reports of 285 cities in China provide the sample data. The data period was scanning from 2003 to 2020. Other data sources included several official national statistical documents like the China Urban Construction Statistical Yearbook, the China Urban Yearbook, and the China Statistical Yearbook. After the disruption, all minor indicators are adjusted to the constant price in 2003 as per the provincial price index.

#### 3.2.1. Dependent variable

The total carbon emissions is the dependent variable. To prevent double counting, the annual consumption of various types of energy in each city was subtracted from the input and loss of energy processing and conversion process and industrial production as raw materials, and the net consumption of 285 cities was obtained. As per the regulations stipulated in the 2006 IPCC Guidelines for National Greenhouse Gas Inventories issued by the IPCC Panel on Climate Change (IPCC), carbon emissions from fossil fuel combustion are projected from the amount of fuel burned and default emission factors. When calculating urban carbon emissions as explained by Zhang et al. (73), the consumption of three energy sources is mainly considered. These energy sources are liquefied petroleum gas (*LPG*) represented by *LCO*_2_, natural gas (*NGas*) represented by *NCO*_2_, and electricity (*ET*) represented by ECO_2_. Below is the formula for calculating the total carbon emissions:


(5) $$\begin{array}{r} \begin{array}{r} CO_{2}=LCO_{2}+NCO_{2}+ECO_{2}=\sigma_{1}LPG+\sigma_{2}NGas \end{array}\;\\ \begin{array}{r} +\sigma_{3}(k\times ET) \end{array} \end{array}$$

Where σ_1_ represents the carbon emission factor of LPG with a value of 3.1013 kg/m^3^; σ_2_ stands for the carbon emission factor of *NGas* with a value of 2.1622 kg/m^3^; σ_3_ represents the carbon emission factor of the coal-fired fuel chain, equal to 1.3023 kg/kW carbon emissions; κ is the ratio of coal-fired power generation to total power generation.

#### 3.2.2. Key independent variable

In this study, we chose *DZP*_*it*_ as the key dependent variable, which is a dummy variable that describes the application status of development zone policies in pilot cities. When *DZP*_*it*_= 1, it means that the ith pilot city began to implement the development zone policy in t, and the rest of the cases are 0. Specifically, from 2008 to 2012, a total of 113 cities implemented the development zone policy. After 2013, by 2020, a total of 7 cities implementing the development zone policy will be added, for a total of 120 pilot cities. Due to the non-uniform nature of policy timing points, multi-period DID was used for correlation analysis.

#### 3.2.3. Control variables

Studies conducted previously by other researchers (74, 75), explain that to prevent omission of small variables related to the level of total carbon emissions, we need various control variables. For this study, the control variables are: (1) Foreign direct investment (*FDI*): expressed by the ratio of foreign direct investment to GDP. (2) Urban rate (*UR*): expressed by the ratio of the urban population to the total population of the city (3) Per capita GDP (*PGDP*): expressed by logarithmic form of per capita GDP; (4) Finance Decentralization (*FD*): expressed by the form of the ratio of fiscal expenditure to fiscal revenue; (5) Population density (*DENSTY*): expressed by logarithmic form of the ratio of the total urban population to the urban administrative area; (6) Industrial upgrading (*IU*): expressed by the ratio of the total industrial value of the secondary industry to GDP and the ratio of the total output value of the tertiary industry to GDP.

This paper gives relevant statistical descriptions of independent variables, dependent variables and control variables. The number of valid samples is 5,130, and the mean, standard deviation, minimum and maximum values of each variable are statistically described. The specific details are shown in Table 1.

**Table 1:** Descriptive statistics.

**Variables**	**Observations**	**Mean**	**S.D**.	**Min**	**Max**
*TCE*	5,130	25.988	23.617	1.529	230.712
*DZP*	5,130	0.472	0.499	0.000	1.000
*lnPGDP*	5,130	10.252	0.837	7.545	13.056
*lnDENSITY*	5,130	5.726	0.916	1.547	7.923
*FD*	5,130	2.814	1.869	0.649	18.399
*IU*	5,130	0.933	0.512	0.094	5.348
*UR*	5,130	0.495	0.174	0.078	1.000
*FDI*	5,130	0.022	0.025	0.000	0.376

## 4. Empirical analysis

### 4.1. Benchmark regression test

It is important to note the regression analysis is conducted on the primary variables first then other overall variables in the results of the regression analysis are shown in Table 2. From column (1), it is found that the coefficient of the key variable *DZP* is −1.390. The coefficient reaches a significant level of 5%, indicating that the implementation of the development zone policy is conducive to the reduction of the total carbon emissions of the pilot cities. Column (2) of the table indicates that with the upsurge of the control variables, the coefficient of *DZP* and the significance test do not change considerably. These results mean that control variables have the very minimal effect on the total carbon emissions of the pilot cities. This conclusion is consistent with Gao et al. (76), whose research on the impact of development zones on carbon emissions, and the establishment of development zones has a positive impact on the city's carbon emission performance. Table 2 also shows that in the control variables, *FD, FDI* and the total carbon emissions of the pilot cities have the positive relationship of change. On the other hand, *lnPGDP, lnDENSITY, IU, UR* and the total carbon emissions of the pilot cities have an inverse relationship of change relating to the total carbon emissions of the pilot cities.

**Table 2:** Benchmark regression results.

**Variables**	**TCE**	
	**(1)**	**(2)**
*DZP*	−1.390^**^	−1.057^*^
	(0.603)	(0.553)
*lnPGDP*		−0.469
		(1.346)
*lnDENSITY*		12.252^***^
		(4.387)
*FD*		−0.651^***^
		(0.156)
*IU*		2.024^***^
		(0.739)
*UR*		8.132^**^
		(3.497)
*FDI*		−30.376^***^
		(11.613)
City fixed	Yes	Yes
Year fixed	Yes	Yes
Observations	5,130	5,130
R-squared	0.966	0.968

Robust standard errors in parentheses, ^***^, ^**^, and ^*^ indicate significance at the 1, 5, and 10% levels, respectively.

### 4.2. Robustness test

Several robustness tests were necessary to demonstrate the robustness of the results gotten from the regression tests on the core results. These robustness tests included PSM-DID model evaluation, endogeneity test, dynamic effect analysis, parallel trend test, placebo test, and other test removing municipalities that can prove robustness of regression analysis. Based on the results from a series of analysis the core results have strong robustness. In other words, the effect of development zone policies in pilot cities on the total carbon emissions is very significant.

#### 4.2.1. Parallel trend test

According to the parallel trend test of carbon emissions related to special economic zones by existing scholars (50), he believes that after the establishment of new special economic zones, the trend of per capita carbon emissions is consistent with that before the policy impact. This means that the assumption of parallel trends is valid. After the impact of relevant policies, carbon emissions show a significant downward trend, which further indicates that the establishment of new special economic zones will have a negative impact on the carbon emissions of cities. As a kind of special economic zone, whether the development zone has the same parallel trend of carbon emissions. This paper also conducts a parallel trend test for this problem. The test results are shown in the following figure.

**Figure 2:**
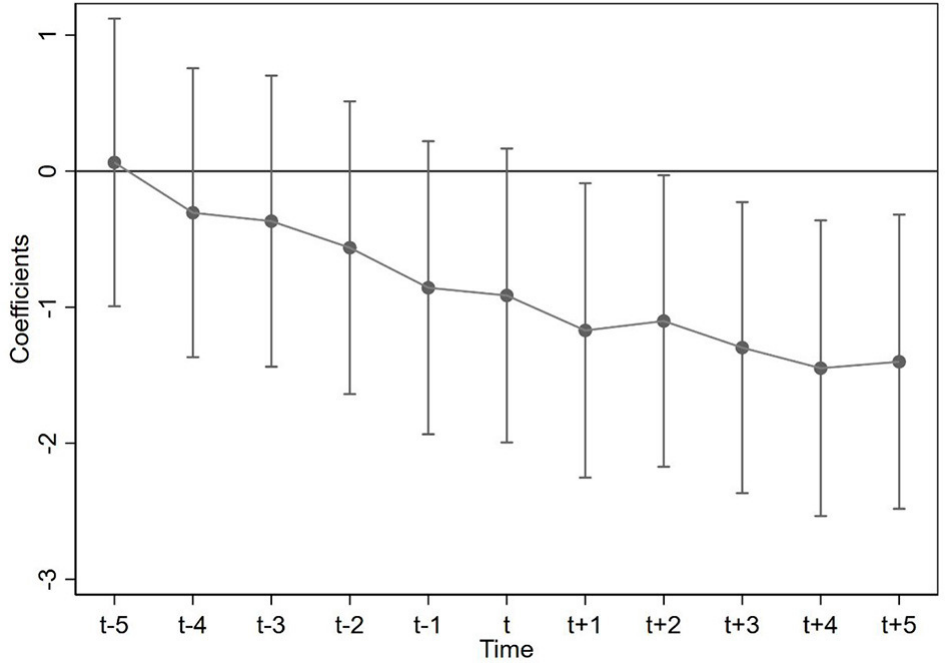
Parallel trend test chart.

The horizontal axis in Figure 2 represents the years before and after the implementation of the development zone policy. On the other hand, the vertical axis on the same figure depicts its correlation coefficient. For example, *t*-4 represents the 4th year before the policy implementation, and *t*+4 represents the 4th year after the policy implementation.

In Figure 2, from year *t*-5 to year *t* of the policy execution, the coefficients all contain 0 values within the 95% confidence interval. These results indicate that the development zone policy when implemented and not enforced in the cities have the same time trend and that the effect of the policy on the carbon emissions is insignificant From year *t*+1 to year *t*+5 of the policy execution, all coefficient values in the 95% confidence interval are < 0. Likewise, this nature shows that enforcing the development zone policy in pilot cities and non-enforced cities share the same trend. Consequently, carbon emissions decreased significantly during this time period because of the enactment of the development zone policy in the cities. Thus, this test holds and satisfies the parallel trend hypothesis of this study. Over the period of 5 years after implementation of the development zone policy in these cities, there was a gradual decrease of the coefficient value for key independent variable. Before the policy was implemented, the coefficient value was smaller showing that the implementation of the development zone policy has a significant inhibitory effect on the total carbon emissions.

#### 4.2.2. Placebo test

**Figure 3:**
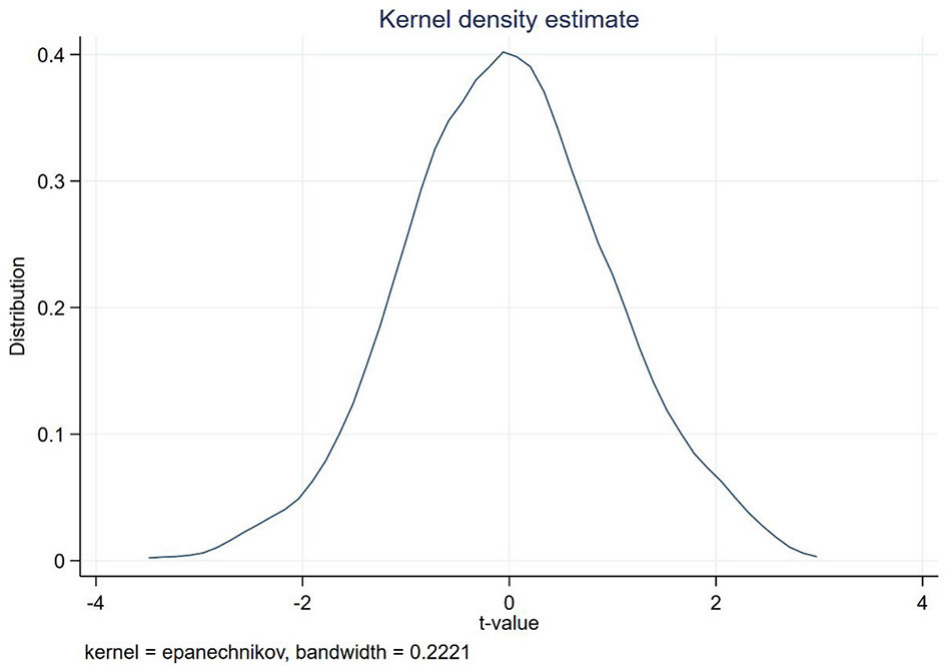
Placebo test chart.

Referring to the placebo test conducted by scholars (77) on the impact of national independent innovation demonstration zones on urban carbon emissions, according to the number of pilot cities launched each year, the same number of cities were randomly selected as the experimental group to construct a dummy variable baseline model. Perform 1,000 and 2,000 repeated regressions on the data. This article discusses how development zone policies affect carbon emissions. We adopted the placebo test to try and evade situations like sample selection bias (38). Specifically, there were 113 cities from 2008 to 2012, that executed the development zone policy. Later in 2013, 7 cities were added in 2020 year, for a total of 120 pilot cities for the study. Through the period from 2008 to 2020, there are 5,130 – 1,525 = 3,605 data samples in the control group and 113 × 5 + 120 × 8 = 1,525 data samples in all experimental groups. We randomly selected 113 cities that implemented the development zone policy from 2008–2020 to collect data. To collect data from the second group of cities, we also randomly selected 7 cities that did not implement the development zone policy from 2013 to 2020 as cities that implemented the development zone policy. In total, 120 cities were randomly selected as the treatment group for the placebo experiment. As shown in Figure 3, the vertical axis represents the corresponding *p*-value while the horizontal axis represents the t value of the development zone policy. The overall figure shows that the distribution is roughly centered at 0. Most of the *p*-values are >0.1, and the absolute value of the corresponding *t*-value is < 2. Results from this test analysis support that the effect of our development zone policy on the total carbon emissions is not affected by the omitted variables.

#### 4.2.3. PSM-DID assessment

During this study, we relied on research methods of existing literature and use PSM-DID to conduct robustness tests. This approach allowed us to avoid systematic differences among cities that do not implement policies and cities that implement policies by reducing the estimation bias of the key variable *DZP*. The primary stages are as follows: first, conduct logit regression on the control variables in the baseline model to get propensity scores; After that, use the non-pilot cities with the most alike propensity scores as the paired objects for the pilot cities; third, use the DID model to approximate the paired sample cities. As shown in Table 3, from the approximation results, the coefficients before the key variable *DZP* are all negative, from the comparison of the (1) and (2) columns, the coefficient values are very approximative when considering and not considering the control variables. These results indicate that the application of the development zone policy has successfully lowered the total carbon emissions. Conclusively, we hold that the core results have strong robustness.

**Table 3:** PSM-DID regression results.

**Variables**	**TCE**	
	**(1)**	**(2)**
*DZP*	−1.739^***^	−1.693^***^
	(−6.237)	(−6.144)
Control variables	No	Yes
City fixed	Yes	Yes
Year fixed	Yes	Yes
Observations	4,695	4,695
R-squared	0.942	0.945

Robust standard errors in parentheses, ^***^ indicates significance at the 1% level.

#### 4.2.4. Dynamic effect test

Because economic and social development have a certain inertia, the impact on economic operation after the implementation of the policy will not be immediate. It is probable that the impact of applying the development zone policy will be many periods or a one-period lag. For this study we only factor the likely effect of the policy with one-period lag on the total carbon emissions of the city. In essence, we considered the dynamic effect for the research. So, we set the one-period lagged core variable as *LDZP* and re-regressed it in the study with all control variables with one-period lag. Results of these modifications are displayed in Table 4. According to the test data of the core variable *LDZP*, the application of the development zone policy still affects the total carbon emissions in the cities. Through the comparison of Tables 2, 4, it is found that majority of the independent variable coefficients still have high consistency, and their significance is slightly different, and it is obvious that the lagging policy effect is not significant.

**Table 4:** Dynamic test results.

**Variables**	**TCE**	
	**(1)**	**(2)**
*L.DZP*	−1.122^**^	−0.845^*^
	(0.523)	(0.482)
Control variables	No	Yes
City fixed	Yes	Yes
Year fixed	Yes	Yes
Observations	4,845	4,845
R-squared	0.973	0.974

Robust standard errors in parentheses, ^**^ and ^*^ indicate significance at the 5 and 10% levels, respectively.

#### 4.2.5. Endogeneity test

To alleviate endogeneity problems, this paper conducts research using the instrumental variable (*IV*) approach. For the assortment of the instrumental variable, refer to the research ideas as discussed by Zhang et al. (78). Ming Dynasty post stations were the national postal and postal transportation system in ancient times. These stations have unique benefits in logistics transportation and information transmission while simultaneously bringing prosperity to the regional economy and development of the nation. It follows that the layout of post stations and the number of these stations have an effect on the economic development of the region, and may also have a relationship with the existing economic development. Consequently, it conforms to the endogenous principle. It is worth noting that there is no direct relationship between the post stations in the Ming Dynasty and the current carbon emissions. Because the Ming Dynasty post station has a long time span, and there is no reasonable relationship between the Ming Dynasty post station and carbon emissions. In this sense, Ming Dynasty post stations satisfy the principle of exogenous assumptions. Keeping in mind that the Ming Dynasty Post Station is already an existing and unchanging historical data, it cannot be directly inserted into the panel baseline model. To overcome this problem, we introduced the number of modern taxis in the city as an instrumental variable and sets the interactive term of the number of post stations in the Ming Dynasty and, denoted by *IV*. The following formulas are established based on Equations (1) and (2):


(6) $$\begin{array}{r} \begin{array}{r} DZX_{it}=\omega_{0}+\omega_{1}IV+\omega_{2}x_{it}+\gamma_{i}+\gamma t+\varepsilon_{it}TCE_{it} \end{array}\\ \begin{array}{r} =\varphi_{0}+\varphi_{1}{\tilde{DZP}}_{it}+\varphi_{2}X_{it}+{½}_{i}+\nu_{t}+\xi_{it} \end{array} \end{array}$$

Equation (6) is the regression of the first stage. The core explanatory variable is represented by *DZP* represents while the instrumental variable is represented by *IV*; Equation (7) is the final regression result. The *DZP* with a wavy line is a new variable column fitted by the first-stage regression. It combines the instrumental variable and the original core explanatory variable. Table 5 shows the final regression results. From the table, it is clear that the instrumental variable has no direct linkages with the total carbon emissions in the cities. As a result, these results are constant with the exogenous hypothesis. From the table, the absolute value implicated with the core variable *DZP* is small. Nonetheless, the absolute value still maintains a significant effect on the core variable *DZP* at the 1% significance level. In this regard, these results align with the endogeneity hypothesis analysis successfully tested endogeneity using carefully selected instrumental variables. However, these results do not show a direct relationship between the dependent variable and an instrumental variable. At the same time, there is an indirect impact on the dependent variable through the core variable *DZP*.

**Table 5:** Endogenous test results.

**Variables**	**DZP**	**TCE**
	**(1)**	**(2)**
*IV*	−0.002^***^	
	(−2.981)	
*DZP*		−54.703^***^
		(−3.210)
Control variables	Yes	Yes
City fixed	Yes	Yes
Year fixed	Yes	Yes
Observations	5,130	5,130
R-squared	0.732	–

Robust standard errors in parentheses, ^***^ indicates significance at the 1% level.

#### 4.2.6. Other related test

Because of the different administrative levels from one city to the next, there is a need to eliminate the differential impact. We achieved it by eliminating the municipalities in the original sample cities, and using the remaining cities as a new sample (the four municipalities of Beijing, Shanghai, Tianjin, and Chongqing were excluded in the new sample). After that, the regression analysis as stipulated in the DID model is used to test data from the new sample. Results of these tests are depicted Table 6. *DZP*, as the primary variable, remains consistent with the core results, while the absolute value of the coefficient is slightly smaller. These results support the fact that application of the development zone policies in cities result in a decline in total carbon emissions in these regions.

**Table 6:** Regression results for samples that remove municipalities.

**Variables**	**TCE**	
	**(1)**	**(2)**
*DZP*	−1.420^**^	−1.080^*^
	(0.603)	(0.554)
Control variables	No	Yes
City fixed	Yes	Yes
Year fixed	Yes	Yes
Observations	5,058	5,058
R-squared	0.954	0.957

Robust standard errors in parentheses, ^**^ and ^*^ indicate significance at the 5 and 10% levels, respectively.

## 5. Further analysis

### 5.1. Heterogeneity test

According to relevant literature (32, 79), it is found that China's industrial carbon dioxide emissions have different emission standards due to different local policies and environmental requirements. Therefore, it is important to note that development zone policies may have varying effects from one city to the next. This phenomenon is caused by factors like large differences in the resource endowments of cities, different urban scales and different geographic locations. These variances are shown in Table 7, columns (1), (2), and (3) show the effect on the total carbon emissions after enforcing the development zone policy in the eastern, central and western pilot cities, respectively. Based on the analysis, development in the central region is balanced and implementation of the development zone policy at this point causes more effect on the decline of carbon emissions. The eastern region in China is relatively developed and the development zone policy is implemented early. Despite these efforts, the marginal effect of the policy is fading, from the current stage, the policy effect is not ideal. The western region in the country is least developed in terms of economy, science and technology, making it too backward in comparison. Therefore, it is essential to hasten the development of these sectors and eventual implementation of the development zone policy. As shown in the table, the implementation of the policy resulted in the slight increase in urban carbon emissions. Columns (4) represents the impact of the implementation of the development zone policy on the total carbon emissions in small cities, column (5) represents the impact of the implementation of the development zone policy on the total carbon emissions in medium cities and column (6) represents the impact of the implementation of the development zone policy on the total carbon emissions in large cities. The conclusion we made is that the larger the city scale, the more beneficial it is to reduce the total carbon emissions. Columns (7) represents the impact of the implementation of development zone policies in resource-based cities on the total carbon emissions. Column (8) represents the impact of the implementation of development zone policies in non-resource-based cities on the total carbon emissions. Conclusively, the enforcement of the development zone policy in non-resource-based cities has a very obvious effect on lowering the total carbon emissions of the city. Conversely, implementing the development zone policy increases the total carbon emissions in resource-based cities.

**Table 7:** Heterogeneity test results.

**Variables**	**TCE**							
	**Geographical location**			**City scale**			**Endowment of resources**	
	**Eastern**	**Central**	**Western**	**Small**	**Medium**	**Large**	**Resource**	**Non-resource**
	**(1)**	**(2)**	**(3)**	**(4)**	**(5)**	**(6)**	**(7)**	**(8)**
*DZP*	−0.784	−1.933^**^	0.061	0.472	0.539	−2.241^**^	1.130	−2.223^***^
	(0.946)	(0.771)	(1.021)	(0.961)	(0.526)	(0.871)	(0.733)	(0.689)
City fixed	Yes	Yes	Yes	Yes	Yes	Yes	Yes	Yes
Year fixed	Yes	Yes	Yes	Yes	Yes	Yes	Yes	Yes
Control variables	Yes	Yes	Yes	Yes	Yes	Yes	Yes	Yes
Observations	1,818	1,800	1,512	896	1,801	2,397	2,070	3,060
R-squared	0.976	0.953	0.961	0.948	0.976	0.978	0.956	0.974

Robust standard errors in parentheses, ^***^ and ^**^ indicate significance at the 1 and 5% levels, respectively.

### 5.2. SDID test analysis

Possibly, there is a spatial connection between carbon emissions and neighboring cities. Neglecting this spatial correlation may cause inconsistent findings when comparing the theoretical results and actual results of the study. To prevent this inconsistency, the spatial impact of implementing development zone policy pilots and the spatial dependence of carbon emissions were taken into account in the model, and the SDID model was used for analysis. The results are shown in Table 8.

**Table 8:** The regression results are based on the SDID model.

**Variables**	**TCE**	
	**(1)**	**(2)**
*DZP*	−5.804^***^	−5.033^***^
	(1.165)	(1.052)
*W_*T, T*_D*	5.252^***^	4.759^***^
	(1.618)	(1.478)
*W_*NT, T*_D*	−3.888^***^	−3.441^***^
	(1.248)	(1.197)
Control variables	No	Yes
City fixed	Yes	Yes
Year fixed	Yes	Yes
Observations	5,130	5,130
R-squared	0.968	0.970

Robust standard errors in parentheses, ^***^ indicates significance at the 1% level.

Column (1) and column (2) prove that the application of the development zone policy has a spatial effect on the total carbon emissions of neighboring cities. Absolute values of the coefficients of variables *W*_*NT, T*_*D, W*_*T, T*_*D*, and *DZP*, are very large and they all reach the 1% significance level. From column (1), it is conclusive that regardless of whether control variables are considered, the development zone policy has the spatial effect of the total carbon emissions of surrounding cities. On the other hand, the coefficient of *DZP* is significantly negative. In other words, the development zone policies can significantly reduce the total carbon emissions. The coefficient of *W*_*NT, T*_*D* is significantly negative, indicating that the spatial effect of the development zone policy pilot cities reduces total carbon emissions in neighboring non-pilot cities. On the other hand, the coefficient of *W*_*T, T*_*D* is significantly positive, indicating that the spatial effect of the pilot cities of development zone policy increases the total carbon emissions of the neighboring pilot cities. This condition may be implicated with the variances in economic scale, the policy implementation and the administrative levels between pilot cities and non-pilot cities. According to Guo et al. (52), whose research stands there are indeed geographical proximity effects and spatial spillover effects between cities in China. This effect is more pronounced between cities that are spatially closer.

### 5.3. Effect test about upgrading and superposition of the policy

To further improve the accuracy of the study, we factored if the upgrading and superposition of policies will still have the same impact on the total carbon emissions as the core results. The results of the regression analysis are shown in Table 9. The policy upgrading effect of the pilot city's upgrading to the national high-tech zone policy from the provincial high-tech zone policy is Column (1). *DZP_up*1 represents the core variable with its coefficient being −2.891 and a significance level of 5%. These results show that upgrading the high-tech zone policy considerably helps to lessen the total carbon emissions. The second column is the policy upgrading effect of the pilot cities' upgrading to the national-level economic development zone policy from the provincial-level economic development zone policy. *DZP_up*2 represents the core variable and its coefficient is −0.131. These results show that upgrading the economic development zone policy causes a minor decrease in the total carbon emissions.

**Table 9:** Policy upgrade and superposition test results.

**Variables**	**TCE**			
	**P to N**	**P to N**	**N plus N**	**N plus N**
	**(1)**	**(2)**	**(3)**	**(4)**
*DZP_up*1	−2.891^**^			
	(1.426)			
*DZP_up*2		−0.131		
		(0.962)		
*DZP_sp*1			−2.745^*^	
			(1.439)	
*DZP_sp*2				−0.665
				(0.761)
Control variables	Yes	Yes	Yes	Yes
City fixed	Yes	Yes	Yes	Yes
Year fixed	Yes	Yes	Yes	Yes
Observations	3,030	2,881	2,423	2,423
R-squared	0.953	0.948	0.981	0.981

Robust standard errors in parentheses, ^**^ and ^*^ indicate significance at the 5 and 10% levels, respectively.

The policy superposition effect of the pilot cities is Column (3). The superposition effect first applies to the national economic development zone policy and then to the national high-tech zone policy. *DZP_sp*1 represents the core variable. Its coefficient is −2.745 with a 10% significance level. These figures show that the superposition of such policies has a relatively clear lessening impact on the total carbon emissions of the pilot cities. Column (4) is the policy superposition effect of the pilot cities first executing the national high-tech zone policy and then executing the national economic development zone policy. The core variable is represented by *DZP_sp*2, and its coefficient is −0.665. These figures support that the superposition of such policies has a feeble impact on the total carbon emissions in the cities. Contrasting column (1), (2), (3), and (4), shows that the high-tech zone policy has more effect than the economic development zone policy in the cities. Contrasting the two columns (1) and (2), shows that the upgrade of the high-tech zone policy has a more significant and clear impact in terms of reducing the total carbon emissions compared to the upgrade of the economic development zone policy. Comparing the two columns (3) and (4), shows that shifting to a national high-tech zone from a national economic development zone can decrease the total carbon emissions of pilot cities more than shifting to a national economic development zone from a national high-tech zone.

## 6. Conclusions and research prospects

### 6.1. Conclusions

By analyzing the results of the study, five main conclusions can be drawn: first, the implementation of the development zone policy can effectively reduce the total carbon emissions of the city; Second, the development zone policy that lags behind a single cycle can still significantly reduce the total carbon emissions of cities; Third, the implementation of development zone policies for cities, larger cities and non-resource cities in the central region has a better effect on carbon reduction than other cities; Fourth, there is a spatial effect on the impact of development zone policies on carbon emissions. Its spatial effect reduces the total carbon emissions of neighboring non-pilot cities and increases the total carbon emissions of neighboring pilot cities. Fifth, there are upgrading and overlapping effects in the implementation of relevant policies. Whether it is upgrading or superimposing, the carbon reduction effect of the high-tech zone policy is better than that of the economic development zone.

### 6.2. Policy implications

The following policy suggestions are put forward based on the above discussions: First, the development zone policy can effectively reduce the total carbon emissions, which means that the formulation of relevant policies can successfully achieve a balance between the ecological environment and economic development, which is a positive measure toward optimizing the ecological environment. Therefore, developing countries similar to China that face similar environmental optimization in the process of urbanization can vigorously promote the construction of development zones. Second, there is a single-period lag effect in the implementation of the development zone policy, which means that there is a single-cycle buffer time for the implementation of relevant policies. Therefore, the government should fully consider the time inconsistency of the policy and the final policy results when formulating relevant policies. The third suggestion is the selection of development zone policies suitable for local cities while considering the city's scale, resource endowment, and geographical location while promoting the inclusive development of the local cities in a targeted manner to evade the variance in the effect of development zone policies on carbon emissions. The western region of China is comparatively backward in technology. However, this region has abundant natural resources and a large urban area. The country's western development project has been advancing in recent years and it is arguable that the region is more conducive for the development zone policies. Science technology and natural resources are ample in the central region of China, thus it is necessary to choose and implement appropriate high-tech zone policies to coordinate the overall development and balance the requirements of development and economy. In the eastern part of China, there is a high population density, technological development and advanced economic development. Moreover, the majority of cities in eastern China are closer to the sea, hence, development of foreign trade is a primary advantage for these urban areas. With this in mind, local economic and technological development is arguably reliant on the development zone policies. Forth, the local government should try to reduce the impact of the spatial effect in the implementation of the development zone policy in response to the spatial effect among cities. The central government is responsible for strictly checking the policy effect of each region, establishing strict regulated rules, and issuing relevant deployment documents. The local governments are implicated with conducting implementations of the policies to categorize regional responsibilities and interests, further preventing any pollution transfer. Finally, according to the research results, the central government should vigorously support the construction of high-tech economic development zones in local areas as much as possible, take innovation as the driving force for sustainable development, and create a win-win development pattern of economic development and ecological protection.

### 6.3. Research prospects

Some of the limitations discovered during the research of this paper may encourage future research on relating subjects. To begin with, bias may have arose during the calculations of industrial carbon emissions because of the limited data on the subject. The aim is to attain an inclusive measure of the environmental efficiency. Thus, we included industrial carbon emissions as one of the undesirable outputs. Nonetheless, as per the “China Urban Statistical Yearbook”, we can only analyze the carbon dioxide emissions from three energy sources, that is, natural gas, electricity and liquefied petroleum gas. And so, the results can be altered or protracted in the future using newer or alternative data sources. The second limitation is that the endogeneity analysis lacked some sample data for the tests. The instrumental variable consists of the number of stations in a city and the product of the number of taxis. Unfortunately, we only managed to collect data for most cities, while we could not get data from other cities because of the limited data collection methods available and limited research. In other words, in all the 285 sample cities used for this study, there are missing data. It follows that this missing data needs to be collected to facilitate supplementary verification. Lastly, we use the dummy variable *DZP* to represent the core variable because the PITI (pollution source supervision information disclosure index) standard used by the 113 pilot cities from 2008 to 2012 is different from the PITI standard used by the 120 pilot cities from 2013 to 2020. The variance in PITI intensity between the two times is not definitely distinguished in terms of treatment. In future, more unified and reliable standards can enhance the study of the effect of national development zone policy on carbon emissions.

## Data availability statement

The raw data supporting the conclusions of this article will be made available by the authors, without undue reservation.

## Author contributions

YF: conceptualization, methodology, and formal analysis. YG: data curation, writing—original draft, visualization, and investigation. SH: writing—review and editing, supervision, and resources. YZ: software, data, and variables. All authors contributed to the article and approved the submitted version.
